# Coping with Accident Reactions (CARE) early intervention programme for preventing traumatic stress reactions in young injured children: study protocol for two randomised controlled trials

**DOI:** 10.1186/s13063-016-1490-2

**Published:** 2016-07-28

**Authors:** Alexandra C. De Young, Ann-Christin Haag, Justin A. Kenardy, Roy M. Kimble, Markus A. Landolt

**Affiliations:** 1 Centre for Children’s Burns and Trauma Research, Centre for Child Health Research, University of Queensland, Brisbane, Australia; 2 Department of Psychosomatics and Psychiatry and Children’s Research Centre, University Children’s Hospital Zurich, Zurich, Switzerland; 3 School of Psychology and Recover Injury Research Centre, University of Queensland, Brisbane, Australia; 4 Division of Child and Adolescent Health Psychology, Department of Psychology, University of Zurich, Zurich, Switzerland

**Keywords:** Young children, Preschoolers, Screening, Early intervention, Prevention, Trauma, Post-traumatic stress disorder, Randomised controlled trial

## Abstract

**Background:**

Accidental injury represents the most common type of traumatic event experienced by children under the age of 6 years. Around 10–30 % of young injured children will go on to develop post-traumatic stress disorder (PTSD) and other co-morbid conditions. Parents of injured children are also at risk of PTSD, and this is associated with short- and long-term consequences for their children’s physical and psychological recovery. Despite the significance of this problem, to date, the mental health needs of injured young children have been neglected. One reason for this is due to the uncertainty and considerable debate around how to best provide early psychological intervention to traumatised children and adults. To address these gaps, researchers and psychologists in Australia and Switzerland have developed the Coping with Accident Reactions (CARE) programme, which is a two-session early intervention designed to prevent persistent PTSD reactions in young injured children screened as ‘at risk’. Two separate international studies are being conducted to evaluate the effectiveness and feasibility of this programme.

**Methods/design:**

The study design for the two proposed studies will employ a randomised controlled trial design and children (aged 1–6 years) who are screened as at risk for PTSD 1 week after an unintentional injury, and their parents will be randomised to either (1) CARE intervention or (2) treatment as usual. Assessment will be completed at baseline (2 weeks) and 3 and 6 months post-injury.

**Discussion:**

This international collaboration provides an excellent opportunity to test the benefit of screening and providing early intervention to young children in two different countries and settings. It is expected that outcomes from this research will lead to significant original contributions to the scientific evidence base and clinical treatment and recovery of very young injured children.

**Trial registration:**

The Australian study was registered with the Australian New Zealand Clinical Trials Registry (ACTRN12614000325606) on 26 March 2014. The Swiss study was registered with ClinicalTrials.gov (NCT02088814) on 12 March 2014.

**Electronic supplementary material:**

The online version of this article (doi:10.1186/s13063-016-1490-2) contains supplementary material, which is available to authorized users.

## Background

Approximately one in four infants and pre-schoolers is exposed to potentially traumatic events (PTEs) [1]. Young children are dependent on adults to keep their environment safe, as they have few skills to protect themselves and have limited understanding of what is dangerous. As a result, injury (e.g., due to dog bites, burns, falls, road traffic accidents [RTAs]) is a particularly common PTE during early childhood [2]. The subsequent medical treatment can also be traumatic and at times chronic and repetitive in nature [3, 4]. Due to multiple unique factors related to their stage of development (e.g., rapid rate of neurobiological development, limited emotion regulation and communication skills, importance of a protective attachment relationship), young children are arguably a high-risk group for experiencing adverse psychological and physiological outcomes following trauma.

Research with young children has documented prevalence rates between 6.5 % and 29 % for acute stress reactions within the first month following an RTA [5] or a burn injury [6] and a post-traumatic stress disorder (PTSD) rate of 10 % 6 months post-burn [3]. Research has also shown that young children can develop depression, separation anxiety disorder, oppositional defiant disorder and specific phobias following a burn, and these disorders are highly co-morbid with PTSD [3]. The majority of children are resilient or experience only transient distress following trauma. However, if left untreated, trauma reactions can follow a chronic and debilitating trajectory for approximately 10–13 % of children [3, 4] and may have serious ramifications for physical recovery [7] and psychosocial and biological development [8].

Parents play an important role in how well young children respond to a traumatic event. Approximately 25–45 % of parents also experience clinically elevated levels of acute stress, PTSD, anxiety and depression within the first 6 months of their young child’s injury [9, 10]. Young children have a limited range of skills to communicate or cope with pain or strong emotions, making them highly dependent on their parents to help them feel safe and secure and to regulate their emotions. It is therefore not surprising that researchers have found parental distress to contribute to the development and maintenance of trauma symptomatology in injured children [10, 11].

The above-mentioned findings provide a strong rationale for providing early psychological intervention programmes that prevent or minimise persistent traumatic stress reactions and other psychopathologies for both children and their parents. Early identification of those at risk of poor outcomes is important, considering that very few children who develop PTSD receive access to appropriate psychosocial services. Even when individuals are offered or directly referred to intervention programmes, uptake and engagement are typically poor, with high rates of early treatment termination [12]. Owing to the common misconception that all young children are resilient to the effects of trauma or misassumptions that emotional and behavioural changes are due to stage of development rather than to the trauma, it is even more unlikely that infants and pre-schoolers will receive the necessary intervention for PTSD if it is not identified during routine medical care or follow-up medical appointments. Taken together, it is clear that early identification and interventions for preventing the development of persistent post-traumatic stress symptoms (PTSS) after childhood trauma are of considerable public health significance.

To date, the majority of research has been focused on treatment of chronic PTSD rather than on early intervention, and many unanswered questions remain in both the adult and child literature regarding who should receive early intervention, as well as the optimal time frame, content and length of early intervention [13]. Currently, one of the most debated issues in the treatment of PTSD is deciding on what is the optimal time frame for providing interventions. The issue is that we know that the majority of individuals are resilient following trauma or experience elevated distress during the acute period but recover within the first few months without needing professional help. Psychological debriefing is an intervention provided in the immediate aftermath of trauma. However, it has created controversy in the trauma literature, as it continues to be widely used despite limited evidence that it is effective at reducing the incidence of PTSD [14]. Of concern is that researchers have found that it may interfere with the natural recovery process [14, 15].

Current guidelines therefore recommend a period of ‘watchful waiting’ or monitoring and screening for risk before providing formal psychological treatment for PTSD following trauma [16]. Following these recommendations, most research attention is now focused on evaluating screen-and-treat or stepped-care models. Currently, systematic reviews of the adult literature provide the most support for multi-session trauma-focused cognitive behavioural therapy (TF-CBT) interventions provided to at-risk individuals within the first 3 months of trauma exposure [13].

Currently, there is limited and mixed evidence available on the efficacy of providing early interventions following medical trauma. So far, only one early intervention, the Child and Family Traumatic Stress Intervention, appears to be effective at reducing school-age child PTSD diagnoses and symptoms following exposure to PTEs, including injury [17]. Researchers in some studies have found that information-based universal prevention interventions provided within 2 weeks post-accidental injury were associated with reduced child anxiety symptoms at 1 month [18] and 6 months post-injury [19] and reduced parental PTSS at 6 months [18]. The authors of a moderator analysis of the randomised controlled trial (RCT) of the basic Internet-based early intervention developed by Cox and colleagues found that the intervention was most effective when given to children (aged 7–16 years) experiencing high levels of distress soon after the accident [20]. Specifically, children in the intervention group who reported high levels of initial distress experienced a large reduction in PTSS, whereas children in the control group demonstrated an exacerbation in PTSS by 6 months. In those in whom initial distress was not elevated, there were no significant differences observed between groups. Additionally, support has been found for a single-session early intervention reducing depressive symptoms and behavioural problems in a sub-sample of preadolescent children (age 7–11 years) involved in RTAs [21]. Only one study has involved an investigation of a two-session early intervention with injured children under 6 years of age [22]). Unfortunately, the intervention was not found to be effective at reducing the presence of child PTSD, PTSS or behavioural problems.

On the basis of results of their meta-analysis and recent early intervention RCT, Kramer and Landolt [22, 23] recommended that, in future early preventive interventions, a stepped-care approach that targets only children screened as being at ‘high risk’ for PTSD should be used. The intervention should be theory-based, include multiple sessions that involve psychoeducation (for children where developmentally appropriate and for parents), targeted coping skills, parent-child relationship focus and some form of trauma exposure (focused on both the injury and subsequent medical procedures). Methodologically sound RCTs of these interventions are needed and should include a priori power analysis to pre-determine sample size, adequate follow-up assessment using clinical interviews and psychometrically sound measures, blinded assessors, clearly defined sample populations, appropriate control groups, adequate randomisation and treatment fidelity checks.

To address some of the aforementioned gaps in the literature, researchers and psychologists in Australia and Switzerland have formed an international collaboration to develop the Coping with Accident Reactions (CARE) programme, which is a two-session early intervention designed to prevent persistent trauma reactions in young pre-school-aged children screened as at risk for PTSD following an unintentional injury. The two research groups are conducting two separate RCTs concurrently to evaluate the effectiveness and feasibility of the CARE early intervention. The following are the specific objectives of the RCTs:

1. Examine if the CARE intervention is more effective than treatment as usual (TAU) in preventing child PTSD and reducing PTSS, internalising and externalising behaviour difficulties in young children with accidental injuries
2. Examine if the CARE intervention is more effective than TAU in preventing and reducing the development of parent PTSD

The primary hypothesis is that children in the CARE intervention condition will have significantly lower PTSS severity scores at 3 and 6 months post-accident than children in the TAU group. The following are secondary hypotheses: (1) Parents who receive the CARE intervention will have significantly lower PTSS severity scores at 3 and 6 months post-accident than parents who receive TAU; (2) children in the CARE intervention group will have significantly fewer PTSD diagnoses at 3 and 6 months post-accident than children in the TAU group; and (3) children in the CARE intervention group will have significantly fewer internalising and externalising behaviour difficulties than children who receive TAU.

## Methods/design

### Design and procedures

**Fig. 1:**
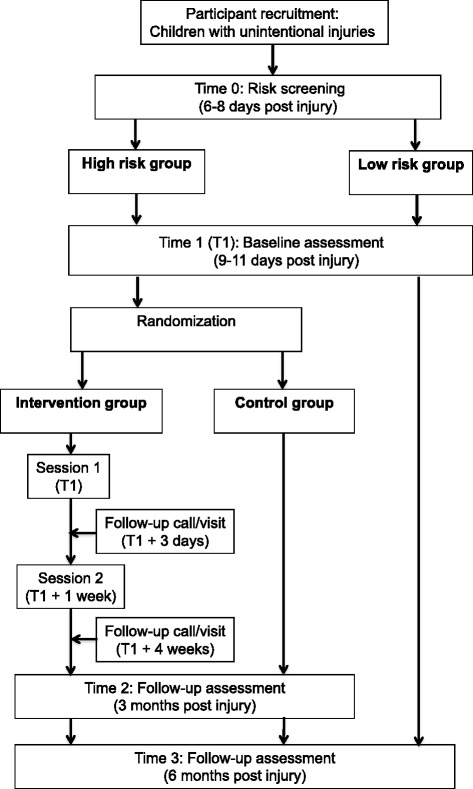
Study flow diagram

The study design for the two proposed research studies is a two-arm, parallel-group superiority prospective RCT comparing the CARE intervention with TAU. The Consolidated Standards of Reporting Trials (CONSORT) guidelines for RCTs will be followed, and the study protocol also adheres to the Standard Protocol Items: Recommendations for Interventional Trials (SPIRIT 2013) (see checklist in Additional file 1). The study involves five stages (see Fig. 1). Stage 1 participant recruitment, will consist of the research team identifying and inviting eligible families to participate in the study and obtaining informed parental consent when the child first presents to hospital. In stage 2, screening, participating parents will be contacted approximately 6–8 days post-accident to complete the screen. Stage 3, baseline assessment and randomisation, will involve the completion of baseline measures by parents of children identified as at risk of PTSD approximately 1–3 days post-screen. Immediately following undergoing baseline assessment, families will be randomly assigned to either (1) the CARE intervention group or (2) TAU. Stage 4, intervention, commences following randomisation. Stage 5, follow-up assessment, consists of 3- and 6-month assessments conducted by trained psychologists blinded to treatment allocation.

### Setting

One RCT is being conducted in Australia at the Lady Cilento Children’s Hospital (LCCH), Brisbane, and the other in Switzerland at the University Children’s Hospital, Zurich.

### Participants

The inclusion criteria for the two studies vary slightly. Participants are invited to participate in the Australian study if the child (1) requires inpatient or outpatient treatment for an unintentional partial- or full-thickness burn injury or is admitted to the LCCH for ≥6 h following an unintentional traumatic injury (e.g., dog bite, RTA, fall) and (2) is aged 1–6 years. Participants meet eligibility criteria for the Switzerland study if the child (1) presents to the University Children’s Hospital for inpatient or outpatient treatment of an unintentional burn and (2) is aged 1–4 years.

Exclusion criteria for both studies are as follows: (1) Parents’ language ability (English in Australia or German in Switzerland) is insufficient to complete measures; (2) child has an initial Glasgow Coma Scale score <12; (3) injury was caused by suspected abuse; (4) child is under the care of child safety; (5) child has a pervasive developmental disorder; and/or (6) expected stay in the paediatric intensive care unit is longer than 1 week.

### Intervention

#### Theoretical underpinnings of CARE intervention

The Pediatric Psychosocial Preventative Health Model (PPPHM) [24] integrative model of Pediatric Medical Traumatic Stress (PMTS) [25] and the relational PTSD model [26] were used to guide the development of the CARE intervention. The PPPHM model incorporates a biopsychosocial competence-based framework and is adapted from the public health prevention framework of universal, selective and indicated. In this model, *universal* represents the majority of families who present to health care settings and appear to be resilient or experiencing distress but coping well. It is recommended that families be provided with general support and information to support their competence, and all children and parents are screened for the presence of risk factors or signs of acute distress. *Targeted* (or *selective*) *interventions* are aimed at families in whom signs of acute distress and/or risk factors are evident. It is suggested that early interventions should be aimed at reducing specific symptoms and monitoring distress over time (e.g., through re-screening at key transition times). The minority of families at the top of this model, *clinical/treatment*, are those experiencing clinically significant, persistent and/or escalating levels of distress and are in need of specialist psychological intervention and support.

The PMTS model describes child and family adjustment across three phases after injury and provides recommendations for assessment and intervention. During phase I, peri-trauma, the goal is to modify the subjective experience of PTEs by providing trauma-informed care and to screen for risk. During Phase II, early, ongoing, evolving, the goal is to screen for risk and to prevent or reduce traumatic stress. Finally, the goal of intervention during phase III, longer-term, is to screen and treat significant traumatic stress.

The relational PTSD model describes the co-occurrence of trauma symptomatology in a young child and the child’s parent [26]. The model proposes that trauma affects not only the child but also the parent and that each member’s symptomatology exacerbates that of the other through dysfunctional parent-child relationship interaction patterns (i.e., withdrawn/unresponsive/unavailable, overprotective/constricting, or re-enacting/endangering/frightening patterns).

#### Description of the CARE intervention

**Fig. 2:**
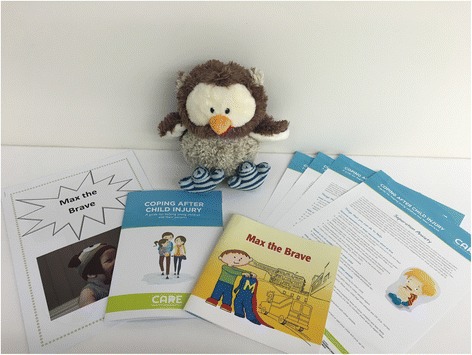
Image of Coping with Accident Reactions intervention materials

The first component of the CARE intervention programme is to provide universal screening to all eligible patients who present to hospital for medical treatment following a traumatic injury. The second component of CARE is a two-session, manualised, targeted intervention [27] for children showing signs of acute distress (i.e., screened as at risk of PTSD) and is delivered by psychologists. The sessions start within the first 2 weeks of the accident (i.e., phase II of the PMTS model) and take approximately 45–60 minutes to complete. The aim of the first session is to (1) provide psychoeducation to help parents understand and normalise both their own and their child’s reactions, (2) provide general coping strategies to prevent or manage acute parent and child distress and (3) provide resources to teach parents how to help their child to talk about and create an accurate story about their accident and medical treatment. Resources developed for the intervention include the information booklets *Max the Brave*, a storybook about a 2-year-old boy who goes to hospital after a burn injury; and Lu Lu, an owl toy and a personal storybook template. Refer to Fig. 2 for a photograph of the English version of the CARE intervention materials.

The aim of the second session (approximately 1 week after session 1 and about 30 minutes in duration) is to (1) monitor child and parent distress levels; (2) educate and normalise how parenting behaviours and the parent-child relationship can change following an accident, help parents identify any unhelpful behaviours (e.g., overprotectiveness, excessive guilt, modelling anxious behaviour) and discuss goals for change; and (3) teach parents how to effectively manage their child’s specific presenting problems.

Two brief follow-up sessions (5–15 minutes in duration) are conducted either in person or over the telephone approximately 3 days after session 1 and 6 weeks post-accident to check progress, briefly review coping strategies and provide referral information if needed. Key components of sessions 1 and 2 are outlined in more detail in Table 1.

**Table 1:** Summary of key components in each session of the Coping with Accident Reactions intervention

Session 1 (9–11 days post-accident)	Session 2 (1 week later)
• Parent’s story about child’s accident and medical treatment • Psychoeducation on parental distress, promotion of coping skills, and activation of resources to manage own distress • Psychoeducation on trauma reactions in young children to help understand and normalise the reactions and to identify signs for ongoing problems • General coping strategies for parents to prevent or manage their child’s distress (All psychoeducation provided orally and in written form.) • Storybook *Max the Brave*; help parents talk to their child about accident-related experiences and to show how the character successfully copes with them • Lu Lu, an owl toy (also introduced in *Max the Brave*) for child’s comfort and to feel brave in scary situations • Instructions for creating the personal storybook (e.g., using photographs, drawings, writing, stickers) about the accident and medical treatment to create an accurate understanding and to provide safe exposure to these memories	• Review of child’s presenting symptoms and concerns over the week • Check parents’ distress level and coping ability and provide referral information if necessary • Check use of *Max the Brave* and Lu Lu, encourage parents to continue doing so • Check if parents are doing the personal storybook correctly, identify any problems and encourage them to continue doing exercises (Book is completed when child’s accident journey has finished.) • Educate and normalise how parenting behaviours and the parent-child relationship can change following a child’s accident; help them to identify any unhelpful behaviours and discuss goals for change • Teach parents how to effectively manage their child’s presenting traumatic stress reactions; teach specific coping strategies for separation anxiety; disobedience, tantrums and aggressive behaviour; sleeping problems; and fear and avoidance

#### Treatment as usual

Treatment as usual consists of standard medical care, including clinical diagnostics and comprehensive, state-of-the-art medical treatment. Depending on the child’s injury, staff members from different disciplines are available for treatment (e.g., surgeons, paediatricians, physical therapists, occupational therapist, social workers). Although not routinely provided, psychological support is also available in Switzerland.

#### Treatment fidelity

The therapists delivering the intervention are clinical psychologists and are either one of the developers of the intervention (ACDY) or have been trained and supervised by the developers of the intervention. Weekly supervision meetings will be held to discuss the delivery of the intervention. Treatment checklists are completed at the end of each session, and adherence will be checked at the completion of the study.

### Measures

The screening measure will be completed approximately 1 week (6–8 days) after the accident. The clinical outcome measures will be completed at about 2 weeks (9–11 days), 3 months and 6 months post-injury (see the recommended SPIRIT flow diagram in Table 2) and were selected on the basis of having demonstrated acceptable psychometric properties and developmental sensitivity.

**Table 2:** Standard Protocol Items: Recommendations for Interventional Trials (SPIRIT 2013) schedule of study recruitment, intervention and assessments

	Study period				
	Enrolment	Allocation	Post-allocation		
Timepoint	-t1	0	t_1_	t_2_	t_3_
Enrolment:					
Eligibility screen	X				
Informed consent	X				
Allocation to risk condition		X			
Interventions:					
CARE Intervention			X		
Assessments:					
Demographic variables		X			
Pediatric Emotional Distress Scale Early Screener		X			
Diagnostic Infant Preschool Assessment			X	X	X
Young Child PTSD Checklist			X	X	X
Child Behavior Checklist for ages 1.5-5			X	X	X
Posttraumatic diagnostic scale			X	X	X
Injury related variables					X

*0* Allocation to the high-risk or low-risk condition occurs via completion of the screener at 6–8 days post- injury. *t1* 9–11 days post-injury, *t2* 3 months post-injury, *t3* 6 months post-injury

#### Screening questionnaire

The Pediatric Emotional Distress Scale-Early Screener (PEDS-ES [28]) is a 21-item caregiver report questionnaire designed to be used to screen for elevated trauma-related behaviour in children aged 2–10 years. Caregivers are required to rate the frequency at which the behaviour has occurred since the trauma on a 4-point Likert scale (0 equal or less often, 1 = a little more often, 2 = much more often, and 3 = very much more often). The PEDS-ES has demonstrated promise as a screening tool for identifying young children (aged 2–6 years) who are at risk of developing PTSD following accidental injury [28]. A score ≥8 indicates that a child is in the at-risk range for developing PTSD.

#### Primary clinical outcomes

The primary clinical outcome, reduction in PTSS severity scores, will be assessed using the Diagnostic Infant and Preschool Assessment (DIPA). The DIPA [29] is a semi-structured diagnostic interview conducted with the primary caregiver of children aged 1–6 years. The DIPA has demonstrated acceptable test-retest reliability [29]. The PTSD module of the DIPA will be used to assess total PTSS severity and impairment in the child.

#### Secondary clinical outcomes

The PTSD module of the DIPA will be used to provide a PTSD diagnosis. The Young Child PTSD Checklist [30] is a 42-item parent-report questionnaire that assesses criteria of the *Diagnostic and Statistical Manual of Mental Disorders, Fifth Edition* (DSM-5), for PTSD in young children. It will also be used to provide a measure of PTSS severity scores and impairment in the child.

The Child Behavior Checklist for ages 1.5–5 years (CBCL/1.5-5; [31]) will be used to assess for changes between the intervention and control groups in total problem behaviour scores and internalising and externalising difficulties. The CBCL/1.5-5 is a 100-item parent-report checklist that measures emotional and behavioural functioning in children ages 1.5–5 years of age. The CBCL/1.5-5 has demonstrated good psychometric properties [31].

The Posttraumatic Diagnostic Scale (PDS; [32]) is a 49-item self-report questionnaire that is used to screen and assess for PTSD in adults. Psychometric evaluation has demonstrated acceptable to excellent internal consistency, good test-retest reliability and acceptable convergent and concurrent validity [33]. The DSM-5 version of the PDS will be used in this study to provide a measure of PTSD symptom severity ratings and level of impairment in functioning among the parents.

#### Additional outcomes

Demographic information (e.g., child and parent ages, socio-economic status, family structure), will be obtained by using questions included in the questionnaire booklet completed at the time of screening. Injury- and hospital-related information (e.g., injury severity, length of stay in hospital) will be obtained from the child’s medical record. The feasibility and acceptability of the intervention will be assessed by looking at (1) participant approach and consent rates, (2) treatment completion, (3) attrition rates, (4) parent satisfaction with the intervention and (5) therapist feedback.

### Sample size

Published data for a sample of 32 high-risk preschool children (had PTSD 1 month post-burn) who received TAU indicate that their PTSS decreased by an average of three symptoms (from 7 [SD = 2.1] to 4 [SD = 3.3]) over 6 months [3]. We assume that, over the course of 6 months, the number of symptoms in participants in the CARE group will have a mean reduction of five (SD = 3) and the number of symptoms in participants in the TAU groups will have a mean reduction of three (SD = 3). These data are based on the level reported in the recovered high-risk children (i.e., those who no longer met PTSD diagnostic criteria at 6 months) [3]. Consequently, we anticipate the mean difference in the number of symptoms between the CARE and TAU groups at 6 months will be two. We assume the SD will be 3 symptoms and an α-level of 0.05. Using a two-sided test, a total sample size of 56 children (28 in each treatment group) is required to complete the study with a power of 80 %. This is equivalent to an effect size of 0.75. We anticipate that approximately 25 % of participants will not complete the 6-month interviews; therefore, we will randomise 76 individual children to get complete primary outcome data for 56 participants.

### Randomisation

A computerised random number generator (https://www.sealedenvelope.com/simple-randomiser/v1/lists) was employed by a researcher not involved in the study to create a randomisation list using blocks of four study participants. Third-party concealment of group allocation was ensured by using a numbered series of opaque, sealed envelopes prepared in advance. Following the baseline assessment, the interviewer opens the sealed envelope to reveal assignment of the participant to the CARE intervention or to TAU.

### Blinding

Randomisation occurs after the baseline assessment; therefore, all interviewers are blinded to treatment condition during the interview. The 3- and 6-month outcome assessments are completed by different psychologists who are blinded to the treatment condition.

### Statistical analysis plan

Prior to investigating treatment outcome, the intervention and control groups will be compared for pre-treatment equivalence by using demographic and baseline measures (e.g., sex, age, injury severity, child PTSS, parent PTSS). If significant differences are found (*p* < 0.001), this will be taken into account by including these variables as covariates in outcome analyses. The association between completers and non-completers and baseline characteristics will be investigated using Fisher’s exact test and Student’s *t* tests. The outcome data will be analysed and reported in terms of statistical significance of differences between groups in change over time, adjusting for the value of the outcome at baseline. To test the association between treatment group and outcome, we will do a linear regression, with the main outcome being change over 6 months and the independent variables being treatment group (CARE or TAU), and the baseline score on the outcome under investigation will be included as a co-variable. When appropriate, repeated-measures analyses will be used to compare the two groups with respect to improvement over time. Generalised estimating equations and generalised linear mixed models will be employed. For sensitivity analysis, if any systematic differences are found according to assessment completion, we will re-run analyses using 3- and 6-month outcome data imputed using multiple imputation techniques. Analyses will be conducted on an intention-to-treat basis, with individuals analysed on the basis of the groups to which they were randomised, regardless of the treatment they received. We define statistical significance as *p* < 0.05.

### Dissemination policy

Outcomes will be published in peer-reviewed journals and will also be presented at relevant national and international conferences. Findings will also be disseminated broadly to participants, health care professionals and the public. If the CARE intervention proves to be effective, implementation in other hospitals is planned.

## Discussion

Currently, research suggests that early preventive interventions may be beneficial for reducing distress associated with childhood injury. However, further empirical evidence is needed to determine optimal timing, length and content needed for interventions to be effective in preventing the development of persistent PTSD and co-morbid conditions. In particular, despite infants, toddlers and pre-schoolers being a particularly at-risk population, to date, the mental health needs of this group are under-recognised and there is very little evidence available to inform research and clinical practice with this population. Health service providers need to become better skilled at detection and treatment of post-traumatic stress reactions in young children.

This international collaboration therefore is an excellent opportunity to test the potential benefit of screening and providing early intervention to young injured children in two different countries and settings. Screening for risk is important because the majority of children, particularly those treated by outpatient hospital services, are not routinely identified and provided with psychological support options. Routine screening has the potential to identify families that are unlikely to engage in mental health treatment as well as improve the efficiency and cost-effectiveness of hospital services. Provision of psychoeducation and coping skills to promote resilience and recovery has the potential to prevent the development of chronic PTSD and co-morbid disorders (that could be later misdiagnosed and mistreated, such as medication for attention-deficit/hyperactivity disorder) and improve treatment adherence and physical recovery (thus improving clinical efficiency with reductions in treatment and rehabilitation duration). Intervening during early childhood, before problems become entrenched and negatively impact critical early development, has the potential to diminish the burden of disease and dysfunction across the lifespan. Given the high incidence of paediatric injury worldwide, a successful early intervention programme for this at-risk population could have significant implications for the social and economic costs associated with medical trauma and beyond.

It is expected that outcomes from this research will make significant original contributions to the clinical treatment and recovery of very young, unintentionally injured children, adding to the resource and scientific evidence base. The parallel research studies evaluating this intervention in Australia and Switzerland highlight the international significance of this problem and will strengthen the generalisability of our findings. Altogether, the findings will help increase the impact and awareness of this important area internationally.

## Trial status

The Australian study commenced recruitment on 3 June 2014 and the Swiss study did so on 1 April 2014. The recruitment for both trials is expected to be completed by the end of 2016 and final assessments by mid-2017.

## Abbreviations

CARE, Coping with Accident Reactions; CBCL, Child Behavior Checklist; CONSORT, Consolidated Standards of Reporting Trials; DIPA, Diagnostic Infant and Preschool Assessment; DSM-5, *Diagnostic and Statistical Manual of Mental Disorders, Fifth Edition*; LCCH, Lady Cilento Children’s Hospital; PDS, Posttraumatic Diagnostic Scale; PEDS-ES, Pediatric Emotional Distress Scale-Early Screener; PMTS, Pediatric Medical Traumatic Stress; PPPHM, Pediatric Psychosocial Preventative Health Model; PTE, potentially traumatic event; PTSD, post-traumatic stress disorder; PTSS, post-traumatic stress symptoms; RCT, randomised controlled trial; RTA, road traffic accident; SPIRIT 2013, Standard Protocol Items: Recommendations for Interventional Trials; TAU, treatment as usual; TF-CBT, trauma-focused cognitive behavioural therapy

## Additional file

Additional file 1: SPIRIT 2013 checklist: recommended items to address in a clinical trial protocol and related documents. (DOC 256 kb)
